# Expanding the Russian allele frequency reference via cross-laboratory data integration: insights from 7452 exome samples

**DOI:** 10.1093/nsr/nwae326

**Published:** 2024-09-14

**Authors:** Yury A Barbitoff, Darya N Khmelkova, Ekaterina A Pomerantseva, Aleksandr V Slepchenkov, Nikita A Zubashenko, Irina V Mironova, Vladimir S Kaimonov, Dmitrii E Polev, Victoria V Tsay, Andrey S Glotov, Mikhail V Aseev, Sergey G Shcherbak, Oleg S Glotov, Arthur A Isaev, Alexander V Predeus

**Affiliations:** CerbaLab Ltd., St. Petersburg 199106, Russia; Bioinformatics Institute, St. Petersburg 197342, Russia; Department of Genomic Medicine, D.O. Ott Research Institute of Obstetrics, Gynaecology and Reproductology, St. Petersburg 199034, Russia; Genetics and Reproductive Medicine Center “GENETICO” Ltd., Moscow 121205, Russia; Genetics and Reproductive Medicine Center “GENETICO” Ltd., Moscow 121205, Russia; Bioinformatics Institute, St. Petersburg 197342, Russia; Genetics and Reproductive Medicine Center “GENETICO” Ltd., Moscow 121205, Russia; Genetics and Reproductive Medicine Center “GENETICO” Ltd., Moscow 121205, Russia; Genetics and Reproductive Medicine Center “GENETICO” Ltd., Moscow 121205, Russia; CerbaLab Ltd., St. Petersburg 199106, Russia; Metagenomics Research Group, St. Petersburg Pasteur Institute, St. Petersburg 197101, Russia; CerbaLab Ltd., St. Petersburg 199106, Russia; FGBE “Children's Scientific and Clinical Center for Infectious Diseases of the Federal Medical and Biological Agency”, St. Petersburg 197022, Russia; Department of Genomic Medicine, D.O. Ott Research Institute of Obstetrics, Gynaecology and Reproductology, St. Petersburg 199034, Russia; CerbaLab Ltd., St. Petersburg 199106, Russia; Department of Genomic Medicine, D.O. Ott Research Institute of Obstetrics, Gynaecology and Reproductology, St. Petersburg 199034, Russia; City Hospital No. 40, St. Petersburg 197706, Russia; CerbaLab Ltd., St. Petersburg 199106, Russia; Department of Genomic Medicine, D.O. Ott Research Institute of Obstetrics, Gynaecology and Reproductology, St. Petersburg 199034, Russia; FGBE “Children's Scientific and Clinical Center for Infectious Diseases of the Federal Medical and Biological Agency”, St. Petersburg 197022, Russia; City Hospital No. 40, St. Petersburg 197706, Russia; Genetics and Reproductive Medicine Center “GENETICO” Ltd., Moscow 121205, Russia; Bioinformatics Institute, St. Petersburg 197342, Russia

**Keywords:** whole exome sequencing, allele frequency, medical genetics, Russia

## Abstract

Population allele frequency is crucially important for accurate interpretation of known and novel variants in medical genetics. Recently, several large allele frequency databases, such as the Genome Aggregation Database (gnomAD), have been created to serve as a global reference for such studies. However, frequencies of many rare alleles vary dramatically between populations, and population-specific allele frequency is often more informative than the global one. Many countries and regions, including Russia, remain poorly studied from the genetic perspective. Here, we report the first successful attempt to integrate genetic information between major medical genetic laboratories in Russia. We construct RUSeq, an open, large-scale reference set of genetic variants by analyzing 7452 exome samples collected in two major Russian cities—Moscow and St. Petersburg. An ∼10-fold increase in sample size compared to previous studies allowed us to characterize extensive genetic diversity within the admixed Russian population with contributions from several major ancestral groups. We highlight 51 known pathogenic variants that are overrepresented in Russia compared to other European countries. We also identify several dozen high-impact variants that are present in healthy donors despite being annotated as pathogenic in ClinVar and falling within genes associated with autosomal dominant disorders. The constructed database of genetic variant frequencies in Russia has been made available to the medical genetics community through a variant browser available at http://ruseq.ru.

## INTRODUCTION

Next-generation sequencing (NGS) has become a *de facto* standard tool in molecular diagnostics of Mendelian disorders. The advances in the field have been drastic; for example, whole genome sequencing (WGS) screening of all newborns is currently deployed in some countries (https://www.genomicsengland.co.uk/news/public-dialogue-genomics-newborn-screening/). However, despite rapid introduction of NGS into research and clinical practice, currently only ∼42% of all patients with suspected genetic pathology receive a definitive molecular diagnosis from the trio-based WGS analysis (reviewed in [1]). There can be many explanations for the less-than-perfect diagnostic record of NGS-based approaches. However, most researchers agree that our ability to predict the effects of individual variants on human health is currently very limited [2,3], and lags far behind our capacity to detect these variants.

One of the most significant advances of our ability to assess variant effects came from global resequencing projects such as 1000 Genomes project [4], the Exome Aggregation Consortium (ExAC, [5]), Genome Aggregation Database (gnomAD, [6]), and National Heart, Blood, and Lung Institute (NHLBI) TopMed [7]. A simple argument that many variants previously listed as pathogenic are found in healthy individuals in too high a frequency to cause a Mendelian disorder has become the most powerful tool for reducing false positive variant-phenotype associations [5]. To this end, the information about the population allele frequency (AF) is currently broadly used for variant interpretation in clinical practice. All of the modern variant interpretation strategies and guidelines, such as the American College of Medical Genetics and Genomics (ACMG) guidelines [8], Russian variant interpretation guidelines [9], and the Sherloc guidelines [10] use AF in healthy populations to classify variant effects, which becomes especially critical for autosomal dominant (AD) diseases.

While global allele frequency databases remain widely useful, the additional value of population-specific reference databases was recently highlighted. Even in the original ExAC publication, Lek *et al.* have shown that filtering of candidate variants using the maximum allele frequency across populations substantially decreases the number of potentially disease-causing variants observed in an individual exome sample [5]. Recently, a substantial effort of the genomic community has been directed at the creation of more diverse and inclusive populational reference as well as resources covering diverse ethnic groups (e.g. [11]; for review see [12]). In many countries, nation-wide sequencing projects have been conducted, including Genome of the Netherlands (GoNL, [13]) and the Han Genome Database (PGG.*Han*, [14]).

The Russian population, representing over 160 nationalities and many unique sub-populations, remains one of the biggest white spots on the global map of human genomic diversity [15]. Several previous studies focused on investigating the genome-wide variation of the Russian population. These include the pilot phase of the Genomes Russia project [16], an exome-based study of monogenic disease prevalence in 694 patients [17], and a targeted sequencing study of 242 known disease genes in 1658 healthy individuals from the Ivanovo region [18]. In these works, several important aspects of the genome variation in Russian patients have been pinpointed. All of these studies, however, lack in the comprehensiveness of the analysis due to either low sample size (such as in the Genomes Russia project) or a narrow set of analyzed genes [18].

Centralized creation of a population genomic reference could benefit from funding security and uniform approaches to sequencing and analysis; at the same time, it is associated with numerous logistical and other difficulties. Most population references were *de facto* obtained using the aggregation of exome and genome data across multiple sequencing centers. The success of ExAC and gnomAD has proven the efficiency and scalability of this approach [5,6]. At the same time, such integration presents a challenging task due to differences in both sequencing approaches and data analysis methods used in different laboratories [3].

In this study, we report the first successful integration of genetic variation data across three major Russian genetic laboratories. To enhance the reproducibility of the analysis and allow a secure and confidential collaboration between the sequencing centers, we developed a portable and reproducible computational pipeline that can be used locally at any future participant laboratory, and subsequently integrated into the global reference using a pre-set analytical and legal framework. We believe that our approach allows for resource- and time-efficient aggregation of data between sequencing centers and will greatly facilitate the construction of a reliable allele frequency reference for the Russian or any other understudied population.

## RESULTS

### Multicenter approach to the creation of Russian coding allele frequency database

To construct a large-scale dataset of exome-wide genetic variation for the Russian population, we have constructed a pipeline to allow for independent raw data processing by multiple sequencing centers (see Methods for details). This pipeline was used to process exome sequencing data from three sequencing centers located in two major cities of the Russian Federation, Moscow (‘Lab 1’) and St. Petersburg (‘Lab 2’ and ‘Lab 3’). A total of 7452 samples were processed in these three laboratories (4630 in Lab 1, 1737 in Lab 2, and 1085 in Lab 3). Aggregation of variant calls across this set of samples allowed for the more than 10-fold expansion of a previous exome-level study which included 694 samples [17]. The majority of samples came from patients with diagnosed monogenic disorders (5507), while 1945 samples were healthy donors or patient relatives, a subgroup that we extensively used for the disease allele prevalence analysis described below.

Following initial data aggregation and genotyping, the dataset was subjected to extensive sample- and variant-level quality control (see Materials and Methods for more details), leaving 6402 samples and 2 218 871 variant sites. Of these, 336 680 variant sites overlapped with the ones reported in our previous publication [17]. Out of all variants, 74.4% were known (found in the dbSNP v154), and 25.6% (567 540) were novel. In total, 1 532 752 variants (69.1%) were either non-coding or silent coding variants, 627 948 (28.3%) were missense mutations or other moderate-impact variants, and 58 171 (2.6%) variants were putative loss-of-function (pLoF) variants. These numbers correspond to a non-synonymous to synonymous variant ratio of 1.79, which is perfectly concordant with the ratio observed in gnomAD v.4.1 (1.78, https://gnomad.broadinstitute.org/stats). In agreement with previous findings, rare and protein-damaging variants were greatly overrepresented among the novel variants. For example, only 24.9% of non-coding and silent coding variants were novel compared to as much as 43.4% of all pLoF variants. Likewise, 90.9% (2 016 705) of all variant sites were rare (MAF <1% in the total sample) compared to as much as 99.4% (563 875) of novel variants. Furthermore, the majority of variants (1 195 496, or 53.9%) were singletons, i.e. were observed only once in the entire dataset (Fig. S3). Finally, allele frequencies of variants in samples of each of the participating parties showed a near-perfect correlation (Spearman's *p* = 0.996 for Lab 2 and Lab 3, 0.997 between Lab 1 and others). Similarly, no significant differences in allele frequencies of common variants were observed between the healthy and diseased donors (Spearman's *p* = 0.999).

### Analysis of the fine genetic structure of the admixed Russian population

Having constructed and characterized our set of genetic variants, we next went on to compare the obtained allele frequencies to the other data on genetic variation in Russia. As already mentioned above, the only available NGS-based genetic variants dataset comes from our previous study with a 10-fold lower sample size [17]. A comparison of variant frequencies between the obtained dataset and the results obtained in our previous study showed a high similarity in variant frequencies (Spearman's *p* = 0.990). We also made an attempt to compare the allele frequencies in our dataset to a recently published microarray-based dataset provided by Biobank Russia [19]. When shared non-imputed variants (*n* = 12 384) were considered, minor frequencies between the datasets showed a high degree of correlation (Spearman's *p* = 0.976), much higher than between either dataset and the gnomAD non-Finnish European (NFE) population (Spearman's *p* = 0.957 for RUSeq and 0.943 for Biobank Russia). Taken together, these results suggest that the RUSeq genetic variant dataset shows high similarity to other datasets of Russian individuals while being the only publicly available exome-wide AF resource to date.

**Figure 1.**
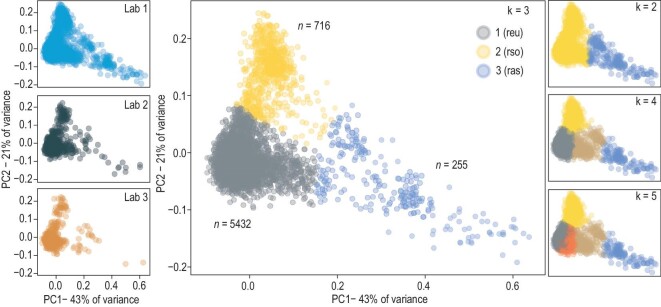
Genetic diversity of individuals in the RUSeq data. Shown are the results of principal component analysis of the genotype data. Each point corresponds to an individual sample. On the left, separate subplots show the results for each participating lab. On the right, dots are colored according to the results of *k*-means clustering with different numbers of clusters (*k*). The middle plot shows the final clustering results used in subsequent analyses. REU, RSO, and RAS correspond to the three clusters (‘heel’, ‘ankle’, and ‘toes’).

In contrast to all previous genomic studies [16–18], our analysis includes a diverse set of admixed samples that can allow us to investigate the fine structure of the present-day Russian population. Indeed, principal component analysis of the individual genotypes showed a substantial level of genetic heterogeneity in the population, with individuals scattered along the first and second principal components. Presence of these clusters could not be explained by place of sample collection (Fig. 1), sequencing platform, disease status, or exome kit (Figs S4–S6).

Given this observation, we went on to group the individuals into clusters using the unsupervised *k*-means algorithm (Fig. 1, right panel), with *k* = 3 being the optimal number of clusters (see Fig. S7). The three clusters significantly differed in size and shape. The first cluster, dubbed ‘heel’, was the densest and contained 5432 (84.8%) samples. The second cluster (‘ankle’) was more sparse and smaller in size, containing 716 (11.2%) samples. Finally, the third cluster (‘toes’) was the smallest and the most heterogeneous, spreading over the first principal component axis (Fig. 1), and comprising 255 (4.0%) individuals. The smallest of these clusters was separated from the other samples even with *k* = 2, and further split into several distinct clusters at larger values of *k*.

Having split the RUSeq individuals into three clusters, we then evaluated the similarity between these clusters. In line with the results of the PCA, the allele frequencies of common variants for the smallest cluster were substantially different from the remaining two clusters (Weir and Cockerham's *F_ST_* = 0.027 and 0.026; Spearman's *p* = 0.91 and 0.92 for largest and second largest clusters, respectively), while the two major clusters were more similar to each other (Weir and Cockerham's *F_ST_* = 0.007; Spearman's *p* = 0.97; Fig. S8). While these values do not indicate profound isolation between these clusters, they suggest possible differences in origins and ancestry of the respective individuals, which we went on to explore in more detail.

**Figure 2.**
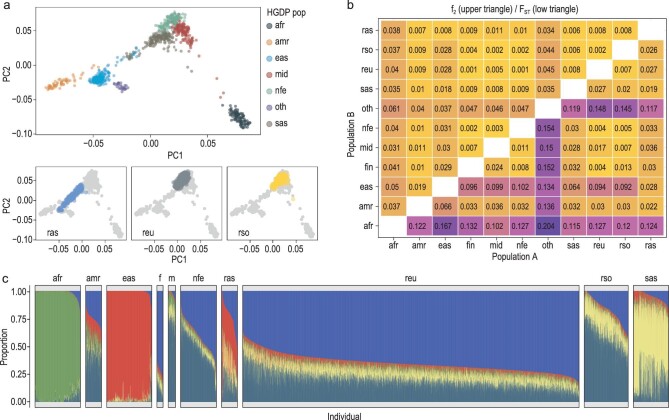
The relationship between the admixed Russian population and major ancestral groups from the Human Genome Diversity Project (HGDP) and 1000 Genomes Project (1KGP). (a) Scatterplot showing samples from HGDP (top) or RUSeq (bottom) plotted in the principal component space built using genotypes of the 402 selected HGDP individuals with approximately even geographical representation (see Methods for details). For plots showing RUSeq individuals, positions of HGDP individuals are represented with gray dots. (b) A heatmap showing pairwise *f_2_* and *F_ST_* values (estimated using the admixtools2 package) between indicated pairs of populations. (c) Barplots showing the results of an ADMIXTURE analysis of unrelated individuals from RUSeq, HGDP, and 1KGP (*K* = 5). The following abbreviations are used for ancestry groups in 1KGP/HGDP: afr—African, amr—American, fin (f)—Finnish, eas—East Asian, mid (m)—Middle Eastern, nfe—non-Finnish European, sas—South Asian, oth—other. reu (RUSeq European), rso (RUSeq Southern), ras (RUSeq Asian) correspond to the three clusters of individuals in the RUSeq data.

To gain deeper insight into the relationship between the admixed Russian population and other world populations, we next integrated the RUSeq data with Human Genome Diversity Project (HGDP) and 1000 Genomes Project (1KGP) datasets and performed the analysis of the resulting joint set of 10 552 samples. As a first step of this analysis, we projected the individuals from RUSeq into the PCA space built using downsampled HGDP data (see Methods for details). The results of this analysis showed that the two largest clusters overlapped with the individuals of European ancestry in HGDP, with the second largest cluster showing a slight shift towards Middle Eastern and South Asian individuals (Fig. 2a, Figs S9 and S10). At the same time, the smallest cluster was spread out between European, South Asian, and East Asian ancestral groups. We next estimated pairwise *f_2_* and *F_ST_* statistics between the three clusters of individuals in RUSeq and eight major groups of HGDP and 1KGP individuals (African, American, East Asian, Finnish, Middle Eastern, non-Finnish European, South Asian, and Other). The results of this analysis corroborated our observations from PCA plots. Thus, the largest RUSeq cluster was closest to Finnish and non-Finnish Europeans (*F_ST_* = 0.004), while the second largest one displayed lower similarity to Finns (*F_ST_* = 0.013) and greater similarity to Middle Eastern individuals (*F_ST_* = 0.007 compared to 0.005 and 0.017, respectively, for the reu cluster). Unlike the other two groups, the smallest cluster showed the greatest affinity towards South Asian (*F_ST_* = 0.019) and East Asian (*F_ST_* = 0.028) populations. Hence, we denoted the three clusters as reu, rso, and ras (for RUSeq European, RUSeq Southern, and RUSeq Asian, respectively). Comparison of the allele frequencies for these groups with data from the Genome Aggregation Database (gnomAD) also confirmed our observations, showing good correlation of allele frequencies between the RUSeq clusters and respective gnomAD populations (Fig. 2b, Figs S11 and S12).

Finally, we performed genetic clustering of the joint HGDP + 1KGP + RUSeq dataset using ADMIXTURE with varying numbers of clusters (*K*). In good concordance with our earlier assumptions, the three groups of individuals in RUSeq differed in their admixture profiles (Fig. 2c, Fig. S13). With *K* = 5, the RUSeq European and Southern clusters had distinct admixture profiles, with the Southern (rso) cluster having greater overall similarity to Southern Eurasia (the Middle Eastern and South Asian groups). The Asian cluster, on the other hand, was characterized by a strong presence of an East Asian component (Fig. 2c).

**Figure 3.**
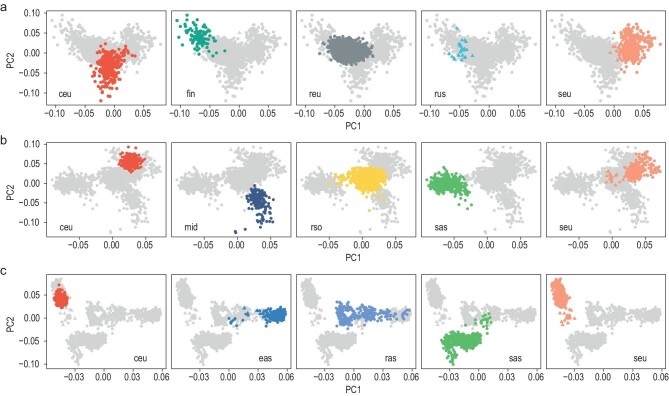
Local mapping of RUSeq individual clusters to reference human populations. (a–c) Projection of individuals from RUSeq and selected populations from the 1000 Genomes project (1KGP) and the Human Genome Diversity Project (HGDP) into a principal component space built using (a) European individuals from 1KGP and HGDP, (b) individuals of European, Middle Eastern, and South Asian ancestry in HGDP, and (c) individuals of European, South Asian, and East Asian ancestry in the HGDP. Principal component analysis of the baseline individuals and projection of RUSeq individuals into the PC spaces was performed using smartpca based on a set of 4419 unlinked high-quality autosomal SNPs. On each subplot, individuals belonging to the indicated population are highlighted. HGDP individuals from subpopulations residing on the territory of Russia (Russian, Adygei (within the Southern European group), Yakut and Hezhen/Nanai (within the East Asian group)) are marked with triangles. The following abbreviations are used for ancestry groups in 1KGP/HGDP: afr—African, amr—American, ceu—Central and Western European, eas—East Asian, fin—Finnish, mid—Middle Eastern, nfe—non-Finnish European, sas—South Asian, rus—Russian individuals, seu—Southern European. The abbreviations reu, rso, ras correspond to the three clusters of individuals in the RUSeq data.

Finally, we attempted to get a glimpse at a finer-grained population structure of our dataset. To this end, we performed a series of projections of the RUSeq samples into PCA spaces constructed using several subsets of HGDP and 1KGP individuals. For the predominantly European cluster reu, we analyzed the respective samples in the context of finer European populations. The results show that the Russian individuals localized in between the three major groups of European populations (Northern, Central, and Western European, Southern European, and Finnish), and showed a nearly perfect overlap with the Russian individuals from HGDP (Fig. 3a, Fig. S14). However, unlike the Russian HGDP group, the much larger RUSeq dataset contained many individuals that were closer to the Southern European populations. Projection of the RUSeq Southern sample cluster rso into a PCA space built from European, Middle Eastern, and South Asian individuals from HGDP showed that the majority of individuals in this cluster were placed between the Southern European and Middle Eastern populations, with a limited number of samples clustered together with the South Asian individuals from HGDP (Fig. 3b, Fig. S15). Again, the RUSeq samples colocalized with the Adygei individuals from HGDP, confirming our hypothesis that the rso cluster is enriched in individuals from the Caucasus region. Finally, analysis of the RUSeq Asian cluster (ras) in the context of European and Asian populations from HGDP confirmed the results of the ADMIXTURE analysis which showed a pronounced East Asian contribution to this group of individuals. Unlike the other two clusters, however, the Asian cluster demonstrated a greater level of diversity, with individuals spread widely between the East Asian, South Asian, and European populations (Fig. 3c, Fig. S16). While the cluster overlapped with the location of Eastern Russia ethnic groups (Yakut and Hezhen) present in HGDP, it contained a distinct group of individuals that did not correspond to any subpopulations present in either 1KGP or HGDP data. While the lack of data on the geographical origin of RUSeq individuals does not allow us to match them to any of the ethnic groups residing in Russia, we believe that these individuals are likely to have at least some of their parents/grandparents of Siberian or Central Asian origin.

### Identification and validation of overrepresented pathogenic and benign variants

Local allele frequency reference compendia are essential for two major applications: (i) to characterize the spectrum of clinically significant variants with high carrier frequency that are located in AR disease genes and can thus be included into early screening programs; and (ii) to identify potentially clinically significant variants (usually located within genes with a dominant inheritance pattern) that are too common in the local population to be interpreted as disease-causing. The expanded set of allele frequencies obtained in this study may aid in solving both of these critically important tasks.

Before identifying overrepresented variants in our data, we examined allele frequencies of pathogenic variants that are known to be present at high frequency in Russia. These include (i) rs113993960 variant in *CFTR* leading to the deletion of a crucial F508 residue of the CFTR protein, and (ii) rs80338939 variant in *GJB2* linked to hearing loss and deafness [20]. In concordance with previous genetic epidemiology studies, both of these variants were present at a very high frequency in our dataset (AF = 0.0063 for rs113993960; AF = 0.0170 for rs80338939). These results validate earlier observations and show that the constructed allele frequency reference accurately captures some of the known clinically significant genetic variation in Russia.

Nearly two dozen prevalent and overrepresented pathogenic variants were reported in the two major sequencing-based studies of the Russian population [17,18]. We first questioned if the variants identified as overrepresented in earlier studies are also confirmed in our dataset. To this end, we selected variants that were reported by Barbitoff *et al.* and Ramensky *et al.* A total of 22 variants were described in these two publications (14 in Barbitoff *et al.*, 2019; and 10 in Ramensky *et al.*, 2021, with 2 overlapping variants). Of these, overrepresentation of 13 variants was successfully validated using the healthy donor subset, and of 16 in the complete set of 6402 samples (Table S1).

We next went on to identify all variants in AR disease genes that showed overrepresentation in our dataset compared to the non-Finnish European individuals in gnomAD (see Methods for details). To this end, we computed the binomial overrepresentation *p*-value for all variants that were reported as pathogenic in ClinVar with no conflicting interpretations. Multiple submissions to ClinVar were required to include a variant into the analysis. As a result, 51 high-quality disease alleles were identified as overrepresented in the healthy donor subset (Table 1, Table S2). The most common of these variants included both known high-frequency variants, such as the rs5030654 variant in *PAH* and the rs1555287300 in *ATP7B* linked to phenylketonuria and Wilson's disease, respectively, and variants that have not been previously reported as overrepresented. Notable examples of the latter category include rs554847663 in *OTOG* and rs72474224 in *GJB2* linked to autosomal recessive deafness, as well as the rs104893924 in *SLC26A2* and rs199952377 in *WDR35* causing multiple epiphyseal dysplasia and cranioectodermal dysplasia, respectively.

**Table 1.** Fifteen most commonly known pathogenic variants present at high frequency in RUSeq.

Variant	Gene	gnomAD NFE AF	Allele count	RUSeq AF*	*p*-value	Disease
12:102840493G > A (rs5030858)	*PAH*	0.15%	23	0.68%	3.56E-09	Phenylketonuria
2:151501423G > A (rs549794342)	*NEB*	0.05%	22	0.65%	4.79E-18	Nemaline myopathy
13:51944145G > T (rs76151636)	*ATP7B*	0.13%	19	0.57%	1.53E-07	Wilson disease
13:113118668C > T (rs41286844)	*C8B*	0.19%	18	0.53%	1.11E-04	C8 deficiency, type II
1:56940965G > A (rs36209567)	*F7*	0.10%	18	0.54%	3.09E-08	Factor VII deficiency
11:17574890C > T (rs554847663)	*OTOG*	0.08%	17	0.53%	1.14E-09	Deafness
11:71441401C > T (rs11555217)	*DHCR7*	0.14%	17	0.50%	1.21E-05	Smith-Lemli-Opitz syndrome
10:89222511C > T (rs116928232)	*LIPA*	0.13%	15	0.46%	3.39E-05	Cholesteryl ester storage disease
13:20189473C > T (rs72474224)	*GJB2*	0.13%	15	0.45%	5.74E-05	Deafness
11:89178603G > A (rs61754365)	*TYR*	0.03%	14	0.42%	1.34E-11	Albinism, oculocutaneous
16:3243310A > G (rs28940579)	*MEFV*	0.09%	12	0.35%	1.10E-04	Familial Mediterranean fever
20:54158136G > A (rs114368325)	*CYP24A1*	0.11%	12	0.36%	3.82E-04	Hypercalcemia, infantile
5:149981550T > A (rs104893924)	*SLC26A2*	0.02%	11	0.33%	1.05E-09	Multiple epiphyseal dysplasia
2:19941796A > C (rs199952377)	*WDR35*	0.03%	10	0.31%	1.03E-07	Cranioectodermal dysplasia
6:80201023G > A (rs386834233)	*BCKDHB*	0.04%	10	0.30%	1.15E-06	Maple syrup urine disease

*AF is given with reference to the healthy donor subgroup.

Notably, several rare variants showed very high levels of fold enrichment in Russia. For example, the rs376910645 variant in the *ATP7B* gene was observed three times in the subset of healthy Russian donors (AF = 0.0009). The frequency of this variant in the gnomAD non-Finnish European population is ∼100 times lower, with only one observation across all gnomAD r. 2.1 exome and gnomAD r. 3.1 genome samples. Another example of a highly enriched pathogenic variant is the rs749076525 in the *DHCR7* gene encoding sterol delta-7-reductase (RUSeq AF = 0.0012, fold enrichment versus gnomAD NFE = 67.4). Mutations in this gene cause Smith-Lemli-Opitz syndrome, an autosomal-recessive condition which involves multiple congenital malformations and intellectual disability. Remarkably, one more variant in *DHCR7* was also found to be significantly overrepresented (rs11555217, observed 17 times in the RUSeq healthy donor group). Similarly, two factor VII deficiency-causing variants were found to be overrepresented in the *F7* gene (rs36209567 and rs121964931, RUSeq AF = 0.0054 and 0.0009, respectively), with the latter variant also having a 25-fold enrichment in the Russian population. Taken together, these data suggest that both Smith-Lemli-Opitz syndrome and factor VII deficiency should have high incidence in the Russian population. For the *F7* gene, similar conclusions were made by us earlier using a smaller set of WES samples [17].

Having characterized the spectrum of overrepresented pathogenic alleles in our dataset, we then went on to identify potentially clinically significant variants in highly constrained genes that are present in healthy patients but are not found in gnomAD. We began by identifying known pathogenic variants missing from gnomAD v2.1.1 data that are reported in the ClinVar database. In total, we discovered 172 such variants, with 53 of them located in genes with autosomal-dominant disease inheritance, and 43 being high-confidence variants with multiple submitters (Table S3). In addition to these variants, we also searched for potentially clinically significant variants that are absent from gnomAD but are present in the healthy subgroup. In total, more than 100 putative loss-of-function (pLoF) variants present in healthy patients were identified. Of these, 59 variants localized in genes with a high degree of evolutionary conservation according to gnomAD-derived metrics (pLI, LOEUF) and with known connection to autosomal dominant phenotypes. A total of 45 variants passed the additional manual curation step. Selected variants are listed in Table S3. Among the known and expected pathogenic variants present in our sample, the most notable example is the rs1064793825 variant in *MSH2* possibly causing hereditary colorectal cancer. A carrier subject has not yet displayed any symptoms of the disorder. However, family history of the carrier listed several cases of cancer, suggesting that the disease is likely to manifest in the near future. Furthermore, we discovered two known pathogenic variants that were present in both healthy and diseased donor subgroups. The first variant, rs786205232 (1:110603893C > T), is a missense variant in the *KCNA2* gene responsible for developmental and epileptic encephalopathy 32 (MIM 616366). The disease allele was observed twice in the diseased cohort and once among the healthy donors. A similar distribution was observed for the other variant, rs59285727 (17:44915251C > T) in *GFAP*, linked to the juvenile form of Alexander disease. The latter example is especially noteworthy as the identified mutation is commonly observed in individuals with Alexander disease [21].

Taken together, we described two important categories of genetic variants in our dataset: (i) known pathogenic variants that are present at higher frequency in Russia, and (ii) variants with presumed pathogenic effects that are too common in Russia and/or are present in the subgroup of healthy individuals. Both of these groups of variants can be used in NGS-based assays in Russia to assist variant interpretation in clinical practice. All of the variant frequency data presented here are available to the community of medical geneticists through a web-based variant portal at http://ruseq.ru.

## DISCUSSION

As shown in multiple studies, population-adjusted allele frequency information is useful for efficient filtering of candidate pathogenic variants. For example, filtering of variants by maximum allele frequency across populations rather than global allele frequency helps to decrease the number of candidate pathogenic variants per individual genome [5]. Despite the great availability of global allele frequency information in such resources as gnomAD, many nations and local populations remain poorly represented from a genetic perspective. Russia, being the world's largest country by total land area, has long remained the widest blank spot on the genetic map of the world [15].

Three major studies have addressed the problem of limited genomic information about the Russian population. One of the studies included only healthy donors from different ethnic backgrounds [16]; however, this study suffered from a very low sample size that was not sufficient for clinical purpose; furthermore, the study results were not made publicly available. An earlier study from our group included 694 samples and provided the first exome-wide estimates of disease allele prevalence [17]. Finally, a recent study by Ramensky *et al.* included 1658 healthy controls; unfortunately, this study was based on a targeted sequencing of a small number of genes (242) [18]. Despite the aforementioned limitations, data obtained in previous analyses have already been used for interpretation of clinical significance of variants [22,23] and population genetics analyses [24,25]. Exome-based allele frequencies have been included into several databases, including a database of *BRCA1/BRCA2* gene mutations (https://oncobrca.ru/). A dramatic (more than 10-fold, 7452 compared to 694) increase in the sample size achieved in this study brings the available allele frequency reference for the Russian population to the level of such a project as ESP6500 [26], greatly aiding clinical specialists in variant interpretation in NGS-based assays.

Increase in the sample size allowed us to make a first glimpse into the genetic structure of the admixed Russian population. While the genomes of several ethnic groups residing in Russia have been studied in global and local projects (e.g. [16]), our study is the first attempt to characterize the genetic diversity of the admixed population. Our analysis showed that the Russian population has a high level of diversity, with genetic contributions from European, Central/South Asian, and East Asian groups (Fig. 2). Local mapping of the samples from RUSeq to subpopulations from reference datasets revealed that the Russian individuals of likely European ancestry present as a diverse mixture of Central, Northern, and Southern European populations. At the same time, individuals of the anticipated Central or East Asian origin fill in the space between the reference European, Middle Eastern, and East Asian groups (Fig. 3, Figs S14–S16). Our results of the population structure analysis are also backed up by a more recent study that employed microarray genotyping data and showed the existence of several subpopulations with distinct origins [19]. Existence of distinct clusters imply that different genetic disease risk factors could be present in different geographical regions of Russia, which is well known to the Russian medical genetics community. This finding comes as no surprise, but predicates the need for further expansion and diversification of the dataset.

As described in the previous section, we have successfully cross-validated some of the earlier observations regarding overrepresentation of disease alleles in the Russian population. Out of 22 disease-associated variants that were identified in [17,18] we confirmed overrepresentation of 18 variants (81.8%), and identified many novel pathogenic alleles that have greater incidence in the Russian Federation compared to other European populations (gnomAD NFE group). For several of the identified variants, their increased prevalence in Russia has been noted in gene-level studies [20,27,28]. For other variants, such as the rs554847663 variant in *OTOG*, no previous reports were published. Hence, these variants represent novel candidates that are worth looking into in further genetic epidemiological studies. In addition to the overrepresented variants, we find a limited set of variants in AD genes that are annotated as pathogenic in ClinVar and linked to severe disorders but are present in healthy control individuals in Russia (Table S2). This information is important for clinical interpretation of such variants, especially in the Russian population.

While the current sample size allows for more unbiased conclusions regarding the genetic structure of the Russian population, we can still expect a large number of rare genetic variants in the rest of the population that were not covered by our analysis. Recent studies have shown that mutational saturation can only be achieved by integrating hundreds of thousands of samples [29]. Hence, further aggregation of data from sequencing centers across Russia, sequencing of more healthy donors, and inclusion of patients from distinct regions (such as in the initial design of the Genomes Russia project, [15]) are all required to fully characterize the genetic variation spectrum of present-day Russia.

## METHODS

Detailed description of the methods is available in the Supplementary Materials. Briefly, the study comprised groups of patients that were subjected to NGS-based assays for molecular diagnostics and/or genetic screening at three major private centers located in Moscow (Genetico Ltd., denoted ‘Lab 1’) and St. Petersburg (CerbaLab Ltd. and City Hospital No. 40, denoted ‘Lab 2’ and ‘Lab 3’, respectively). As detailed below, the dataset contained both healthy and diseased individuals, with healthy donors accounting for 26.1% (1945/7452) of all study participants. The ‘diseased’ subgroup included various Mendelian or likely Mendelian and non-Mendelian phenotypes (with the majority of samples having neurological and neuro-muscular disorders (see Fig. S1)); the ‘healthy’ subgroup comprised healthy donors who were sequenced for carrier screening purposes or patients with multifactorial pathologies, such as obesity or type 2 diabetes (individuals with diagnosed or suspected monogenic conditions were excluded from the healthy donor subgroup). Nearly all patients were residents of the Russian Federation or republics of the former USSR. All participants signed informed consent for studies and processing of personal data, including medical history data. The study was performed in accordance with the Declaration of Helsinki.

## DATA AND CODE AVAILABILITY

The source code of the pipeline, including the Dockerfile used to build a custom Docker image, can be found at https://github.com/bioinf/russian_exome_pipeline/. The resulting allele frequency dataset is available through an interactive web browser at http://ruseq.ru/. To access the full VCF file with variant frequencies please contact the authors using the request form available on the website.

## Supplementary Material

nwae326_Supplemental_File
