# Supplementary material for: Expanding the Russian allele frequency reference via cross-laboratory data integration: insights from 7452 exome samples
Source: Natl Sci Rev. 2024 Sep 14;11(10):nwae326. doi: 10.1093/nsr/nwae326 (PMC11533896; doi:10.1093/nsr/nwae326)
Supplement: nwae326_Supplemental_File [file nwae326_supplemental_file.docx]

**Expanding the Russian allele frequency reference via cross-laboratory data integration: insights from 7,452 exome samples**

Yury A. Barbitoff^1,3,4,^**^✉^**, Darya N. Khmelkova^2^, Ekaterina A. Pomerantseva^2^, Aleksandr V. Slepchenkov^3^, Nikita A. Zubashenko^2^, Irina V. Mironova^2^, Vladimir S. Kaimonov^2^, Dmitrii E. Polev^1^, Victoria V. Tsay^1,5^, Andrey S. Glotov^1,4^, Mikhail V. Aseev^1,4^, Sergey G. Shcherbak^6^, Oleg S. Glotov^1,4,5^, Arthur A. Isaev^2^, and Alexander V. Predeus^3,^**^✉^**

1 - CerbaLab Ltd., St. Petersburg, Russia
2 - Genetics and Reproductive Medicine Center “GENETICO” Ltd., Moscow, Russia
3 - Bioinformatics Institute, St. Petersburg, Russia

4 - Dpt. of Genomic Medicine, D.O. Ott Research Institute of Obstetrics, Gynaecology and Reproductology, St. Petersburg, Russia

5 - FGBE "Children's Scientific and Clinical Center for Infectious Diseases of the Federal Medical and Biological Agency", Saint Petersburg, Russia

6 - City Hospital No. 40, St. Petersburg, Russia

**Supplementary Methods**

*Exome sequencing*

DNA for sequencing was extracted either from peripheral blood samples (in the majority of cases) or from tissue samples in FFPE blocks according to the standard protocols. Sequencing was performed using either whole exome (5,418 samples; 72.7%) or clinical exome (2,034 samples, 27.3%) capture kits. The following capture kits were used: Agilent SureSelect Human all exon V7, Agilent SureSelect Human all exon V6 + UTR, TruSeq DNA Exome (Illumina) with the xGen® Exome Research Panel v1.0 (IDT) exome capture solution, Nimblegen (Roche) SeqCap EZ MedExome, Illumina Nextera RapidCapture, Illumina TruSeq DNA Exome, Nimblegen (Roche) Inherited Disease Panel (IDP) v2, and Illumina TruSight One. Exome libraries were prepared as described previously [1,2]. All libraries were sequenced using Illumina HiSeq 2500/4000, Illumina NovaSeq 6000, MiSeq, or MGISEQ 2000. All samples were sequenced using paired-end reads with read length varying from 75 bp to 300 bp.

*Bioinformatic analysis*

For bioinformatic analysis of exome sequencing data, we developed a custom Docker-based analysis pipeline to ease data transfer and enable the usage of each laboratory’s own computing resources (Supplementary Figure S2). This pipeline was developed using the Snakemake language for pipeline development, and a custom Docker image was built using the Genome Analysis ToolKit (GATK) v. 4.2.1.0 base image. The Docker image also included all necessary reference files used by the pipeline.

For read alignment, a faster reimplementation of the BWA mem algorithm [3], BWA-MEM2 [4] was used. Aligned reads were sorted and indexed using SAMtools [5]; duplicate read pairs were marked using the GATK v. 4.2.1.0 [6,7]. Next, base quality score recalibration and indel realignment was performed using GATK. Pre-processed alignments were then used for variant calling with the GATK HaplotypeCaller in the ERC GVCF mode. The source code of the pipeline, including the Dockerfile used to build a custom Docker image, can be found at <https://github.com/bioinf/russian_exome_pipeline/>

GVCF files were then transferred between laboratories and aggregated using the GATK GEnomicsDB engine. Aggregated GVCF files were then used for joint genotyping using GATK After joint genotyping, all sample-level genotypes with a total depth of less than 10 reads were set to missing, and variants with AC=0 were excluded. The variant callset was then annotated using the Ensembl Variant Effect Predictor (VEP) v104 with the RefSeq cache file for the corresponding reference genome assembly. After variant annotation, Variant Quality Score Recalibration (VQSR) filtering approach was applied; variants with truth sensitivity score between 90.0 and 99.6 (for SNPs) and 90.0 and 99.3 (for indels) were marked as medium confidence; variants with sensitivity values > 99.6 (for SNPs) and 99.3 (for indels) were excluded as low-confidence calls. The resulting multi-sample VCF file was used for variant quality control, filtering and statistical analysis (see below).

*Quality control and filtering of samples and variants*

Variant quality control and further statistical analysis of allele frequencies was performed using the BROAD Institute Hail statistical genetics library v. 0.2.63-cb767a7507c8 (<https://hail.is/>). First, sample-level quality control was performed using the built-in Hail functionality. Four main sample-level metrics were used: heterozygous to non-reference homozygous variant ratio (het/hom), transition-to-transversion ratio (Ti/Tv), insertion-to-deletion ratio, and mean per-sample genotype quality (GQ). The following filtering criteria were applied: 1.4 < het/hom < 2.6, 2.1 < Ti/Tv < 2.9; 0.5 < i/d < 1.0. Samples not meeting any of these criteria were removed from further analysis. We also excluded samples with less than 80% of common CDS bases covered at least 10x. In total, 621 samples were removed.

After sample-level QC, we went on to filter out related individuals. To do so, we first selected common (MAF > 5%) variants that had a high (> 99%) variant call rate across all pass-QC samples. Next, we calculated kinship statistic using the built-in Hail functionality. The kinship value was calculated using a subset of biallelic LD-independent (*r^2^* value of 0.1 was used for LD pruning) common (MAF > 0.05) variants. A maximum independent set of unrelated samples was computed using the kinship statistic value of 0.0635, removing additional 429 samples.

*Mapping individuals in RUSeq to the global genetic diversity map*

For the initial principal component analysis of individual genotypes in RUSeq, we performed PCA using the built-in Hail functionality. PCA was performed using a subset of independent (*r^2^* < 0.1) high-quality autosomal genetic variants with MAF > 0.05 in RUSeq (a total of 4419 variants were used). Samples were clustered using the *k*-means clustering algorithm in the space of first 10 principal components. The optimal number of clusters (*k*) was selected using the elbow method and sum of squared errors (SSE) (values of *k* ranging from 1 to 10 were tested). Allele frequencies in each cluster were determined using Hail built-in aggregation functions.

For the analysis involving Human Genome Diversity Project (HGDP) and 1000 Genomes Project (1KGP), we downloaded a combined HGDP+1KHP variant call set in Hail format provided by gnomAD [8]. The same set of 4419 genetic variants was extracted from the data and merged with RUSeq data to create a dataset with 4419 variants and 10552 individuals (948 samples from HGDP, 3202 - from 1KGP, and 6402 - from RUSeq). The dataset was then exported to PLINK binary format and converted to the EIGENSTRAT format using convertf tool in the EIGENSTRAT package v. 7.2.0 [9].

For PCA involving all populations, a subset of HGDP individuals with a more even geographical representation was created by random selection of 62 samples from each of the major ancestry groups (African, American, East Asian, Middle Eastern, European, South Asian), adding the remaining 30 individuals belonging to the other regions. PCA was performed using the resulting set of 402 samples using the smartpca tool in EIGENSTRAT v. 7.2.0 with the lsqproject mode, and all of the remaining 10150 individuals were projected into the resulting space. For analyses involving subsets of HGDP and 1KGP individuals, three additional smartpca runs were performed using the following subsets of samples for the construction of PCA space: (i) all individuals of European ancestry (n = 788), (ii) HGDP individuals of European, Middle Eastern, and South Asian ancestry (n = 506); or (iii) HGDP individuals of European, South Asian, and East Asian ancestries (n = 584). *f_2_* and *F_ST_* statistics for the combined dataset were computed using the admixtools2 (v.2.0.4) package [10].

Genetic clustering was performed on the subset of the joint HGDP + 1KGP + RUSeq dataset containing only unrelated (kinship < 0.0625) samples (6392 - from RUSeq, 2356 - from 1KGP, and 720 - from HGDP). Clustering was done using ADMIXTURE v. 1.3.0 [11] with varying numbers of ancestral populations (*K* = 3, 4, 5, 6, 7, 8, 10, and 12).

For the analysis of allele frequency similarity with gnomAD populations, we computed Spearman’s correlation coefficient (*p*) and the *F_ST_* value (using the Weir and Cockerham method [12,13]) using allele frequency information for the same set of 4419 autosomal markers.

*Identification of clinically relevant high-frequency variants*

To identify high-frequency clinically significant variants, all variants were first annotated with the disease inheritance pattern associated with the corresponding gene (using the OMIM catalog data) and gene-level constraint (loss-of-function variant observed to expected ratio upper fraction, LOEUF) values from gnomAD v2.1.1 Two main classes of clinically relevant variants were then selected for further analysis: (i) known pathogenic variants in autosomal recessive (AR) disease genes with statistically significant overrepresentation in the dataset; and (ii) known or likely pathogenic variants in autosomal dominant (AD) disease genes that were identified in healthy subjects.

In the former case, variant overrepresentation was evaluated by computing a binomial *p*-value as follows:

$$p \sim B(n, N, q)$$

where *n* is the observed alternative allele count in the sample; *N* is the total allele number at a given variant site, *q* is the alternative allele frequency in the gnomAD non-Finnish European population, and *B* denotes the binomial distribution PDF. False discovery rate (FDR) adjustment was performed using the Benjamini-Hochberg method. Variants with FDR-adjusted p-value < 0.05 were selected as significantly overrepresented. Only variants reported as pathogenic in ClinVar v.20210908 with no conflicting interpretations and multiple submitters (30,874 variants in total, with 1,421 present in RUSeq data) were used for overrepresentation testing.

In the latter case, we selected known pathogenic variants and likely disease-causing variants (loss-of-function variants in known disease genes) that were both (a) absent in the global reference datasets (gnomAD v.2.1.1) and (b) present in healthy RUSeq individuals. The list of variants was narrowed down to genes with low (< 0.5) LOEUF value to enrich the list for AD disease genes.

Lists of variants identified as overrepresented and/or uniquely present in the dataset were manually curated to exclude misannotated variants.

**Supplementary Figures**


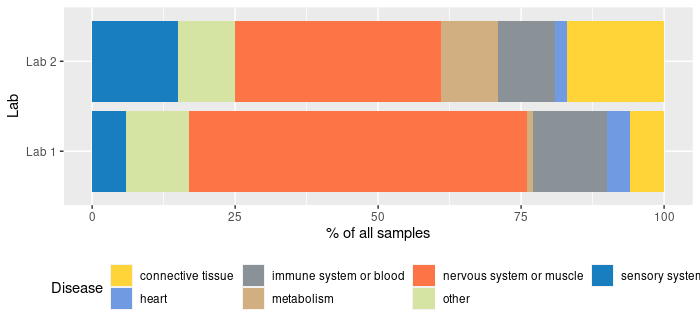


**Figure S1.** Approximate distribution of disease groups in the subset of diseased individuals included into the analysis. Please note that the plot shows proportions of samples rather than absolute numbers due to limited data availability. For Lab 3, most of the samples come from healthy donors. For the rest of the dataset, nosology data are not available.


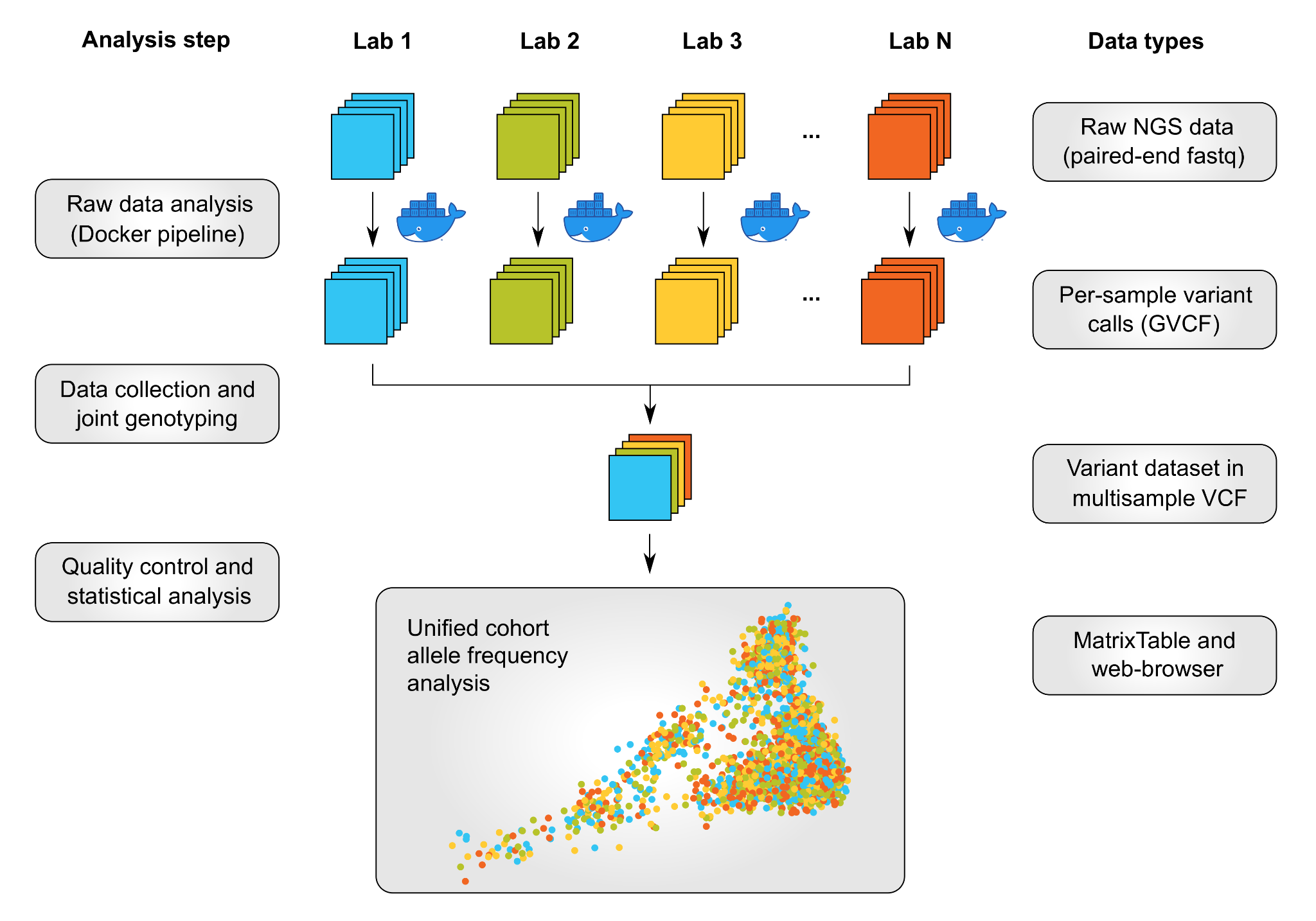


**Figure S2.** A cross-laboratory data integration strategy used for creation of an expanded reference set of allele frequencies in Russian exomes. The first steps of the data analysis, including read alignment and variant calling, are performed using a Docker-based pipeline in each laboratory, avoiding the need for raw data transfer and allowing for a distributed computing process. Variant calls in GVCF format are then aggregated, and joint genotyping is performed to obtain the final variant dataset.

**
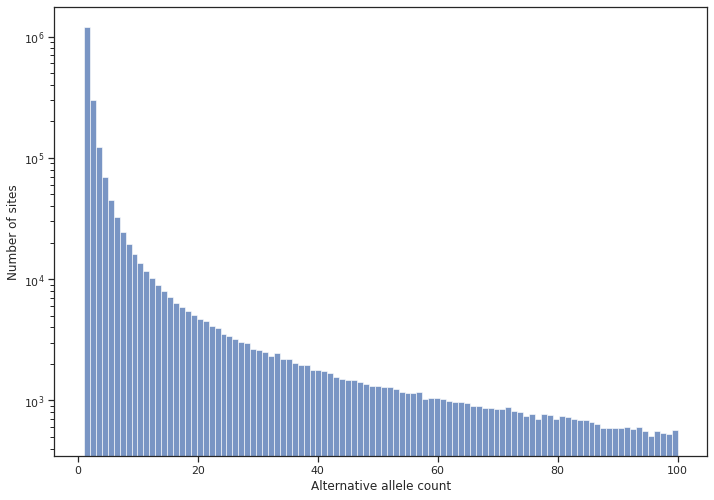
**

**Figure S3.** A site frequency spectrum (SFS) histogram showing the number of variants with different non-reference (alternative) allele count across the dataset. Only sites with AC ≤ 100 are shown.


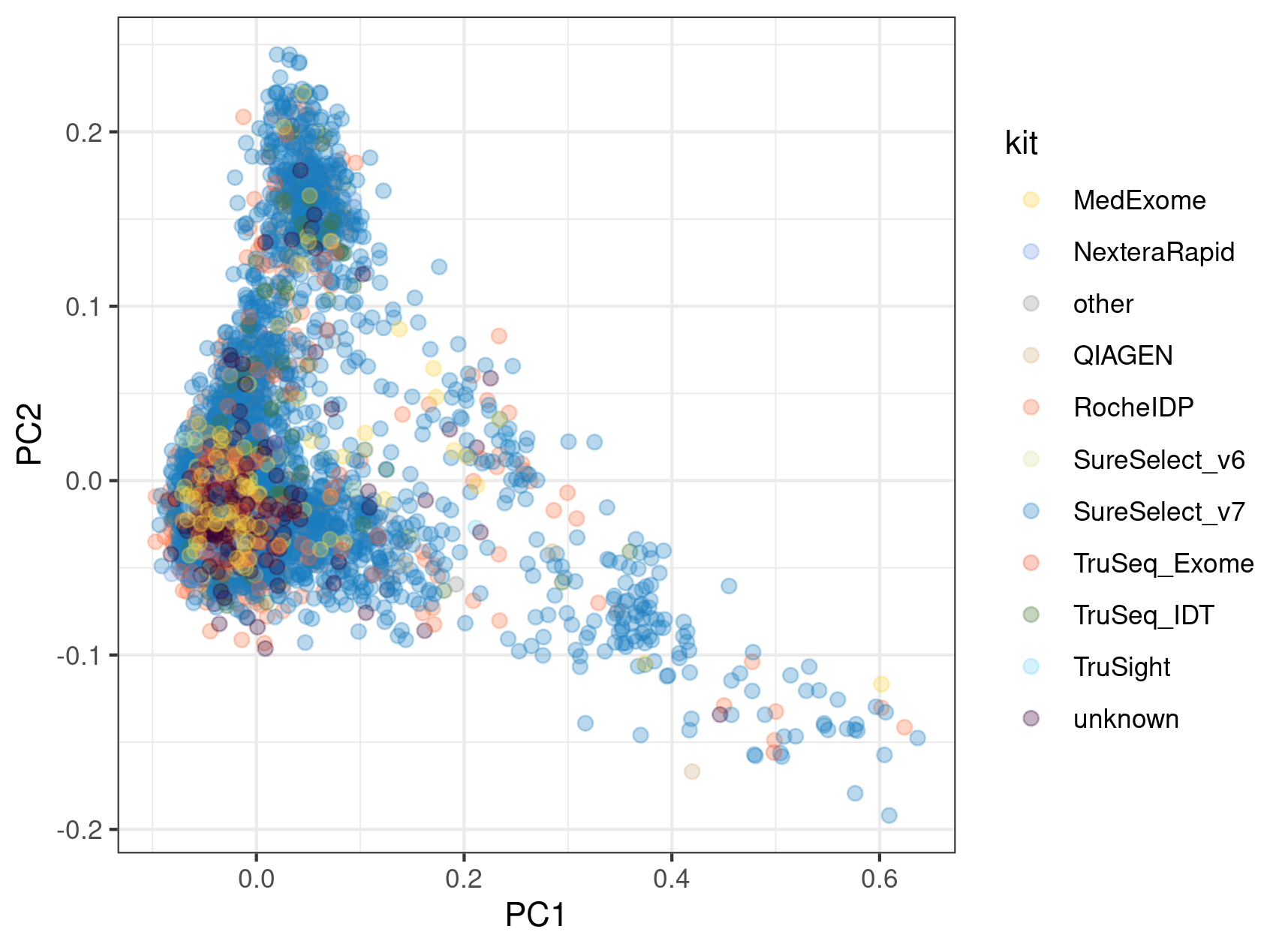


**Figure S4.** A scatterplot showing the results of principal component analysis of individual genotypes coloured by capture probes used for library preparation (A).


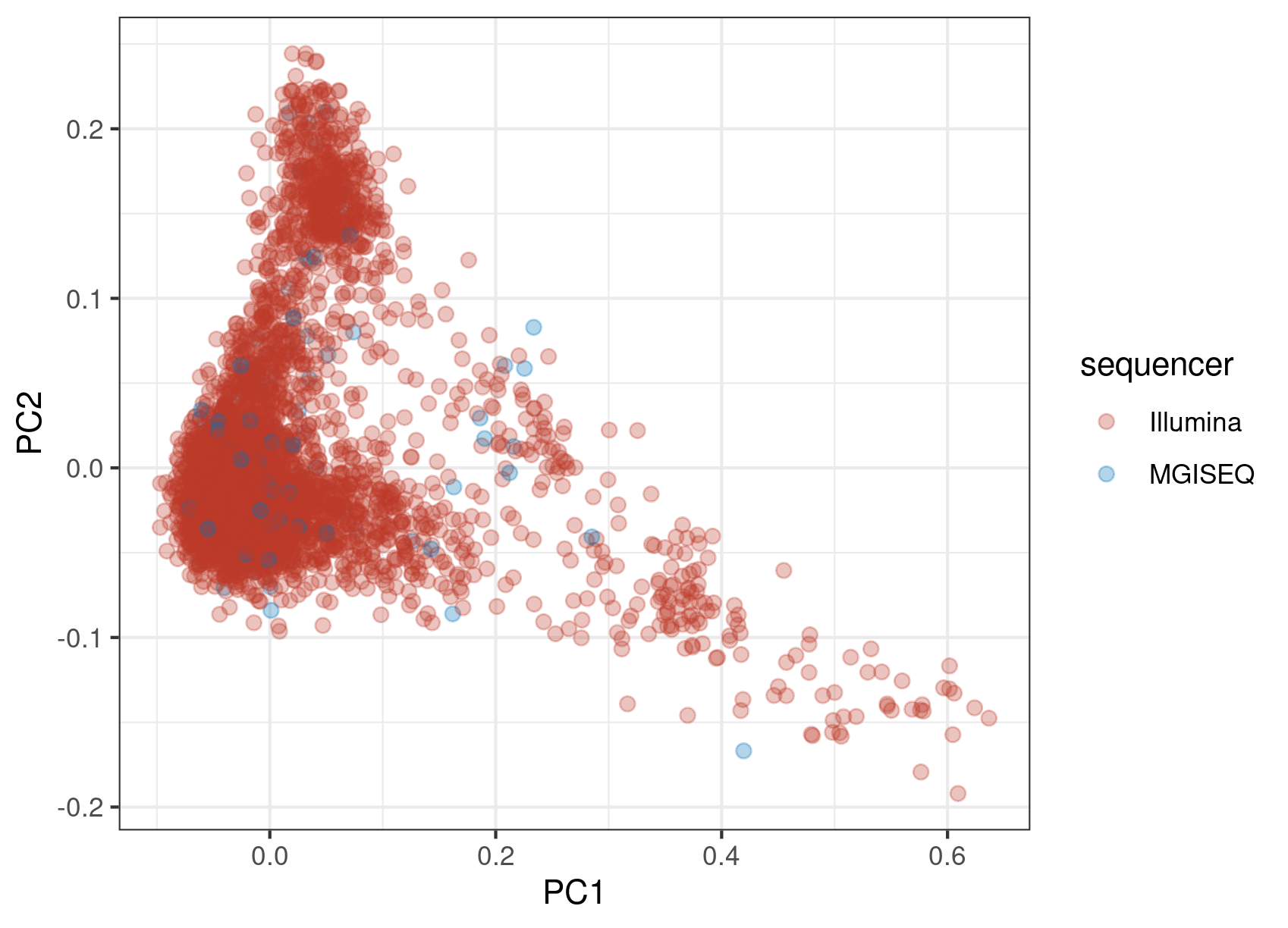


**Figure S5.** A scatterplot showing the results of principal component analysis of individual genotypes coloured by sequencing machine used.


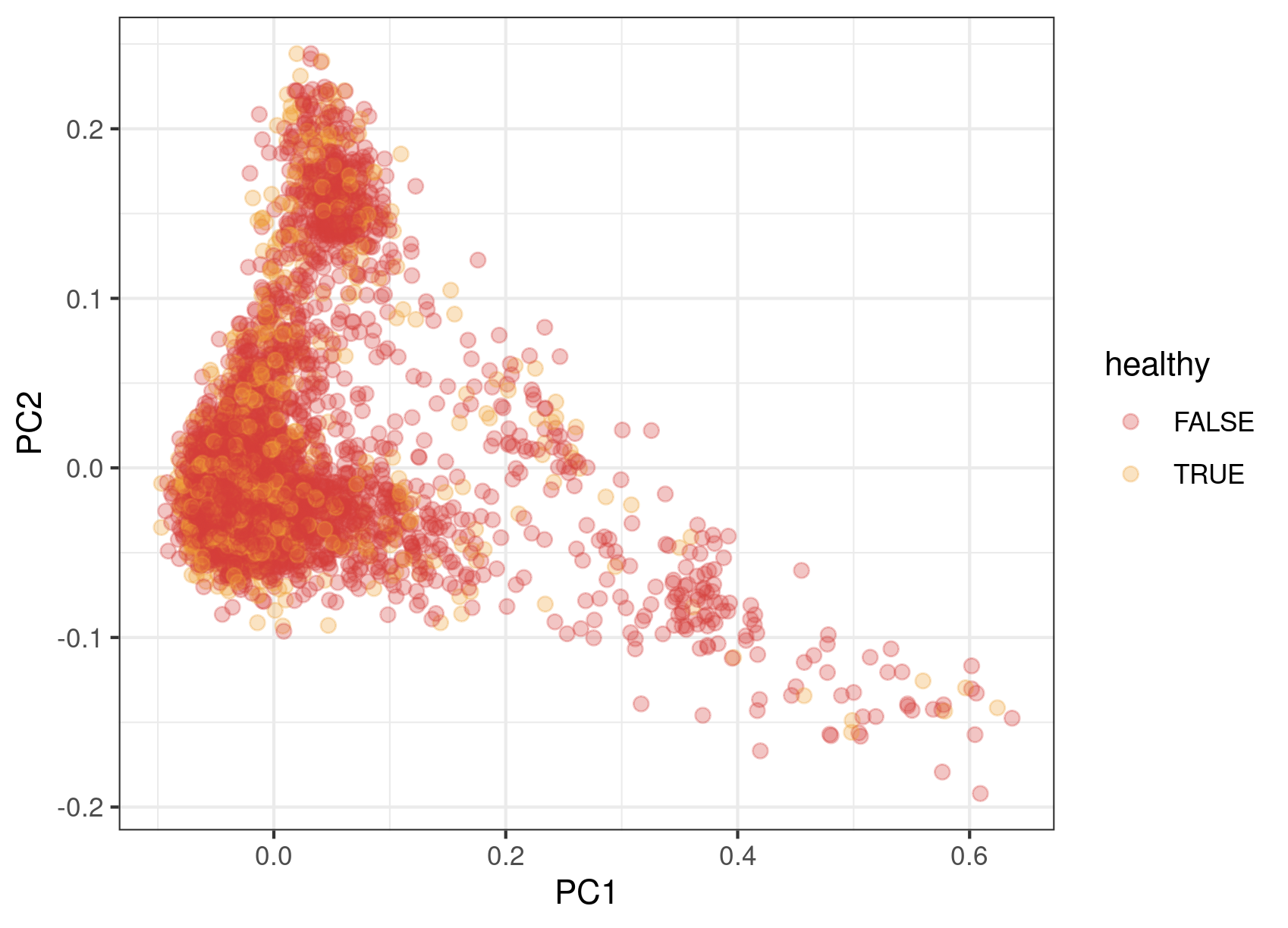
**Figure S6.** A scatterplot showing the results of principal component analysis of individual genotypes coloured by disease status.


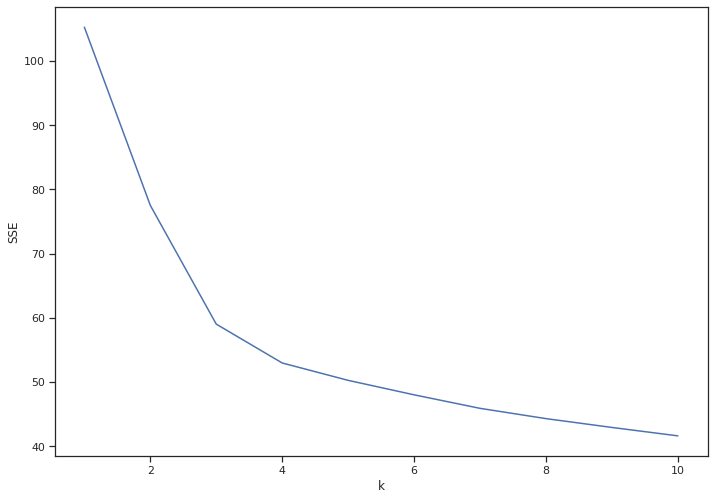


**Figure S7.** An elbow plot showing the relationship between the number of clusters (k) and the sum of squared errors (SSE) in the k-means clustering of the samples in RUSeq.

**
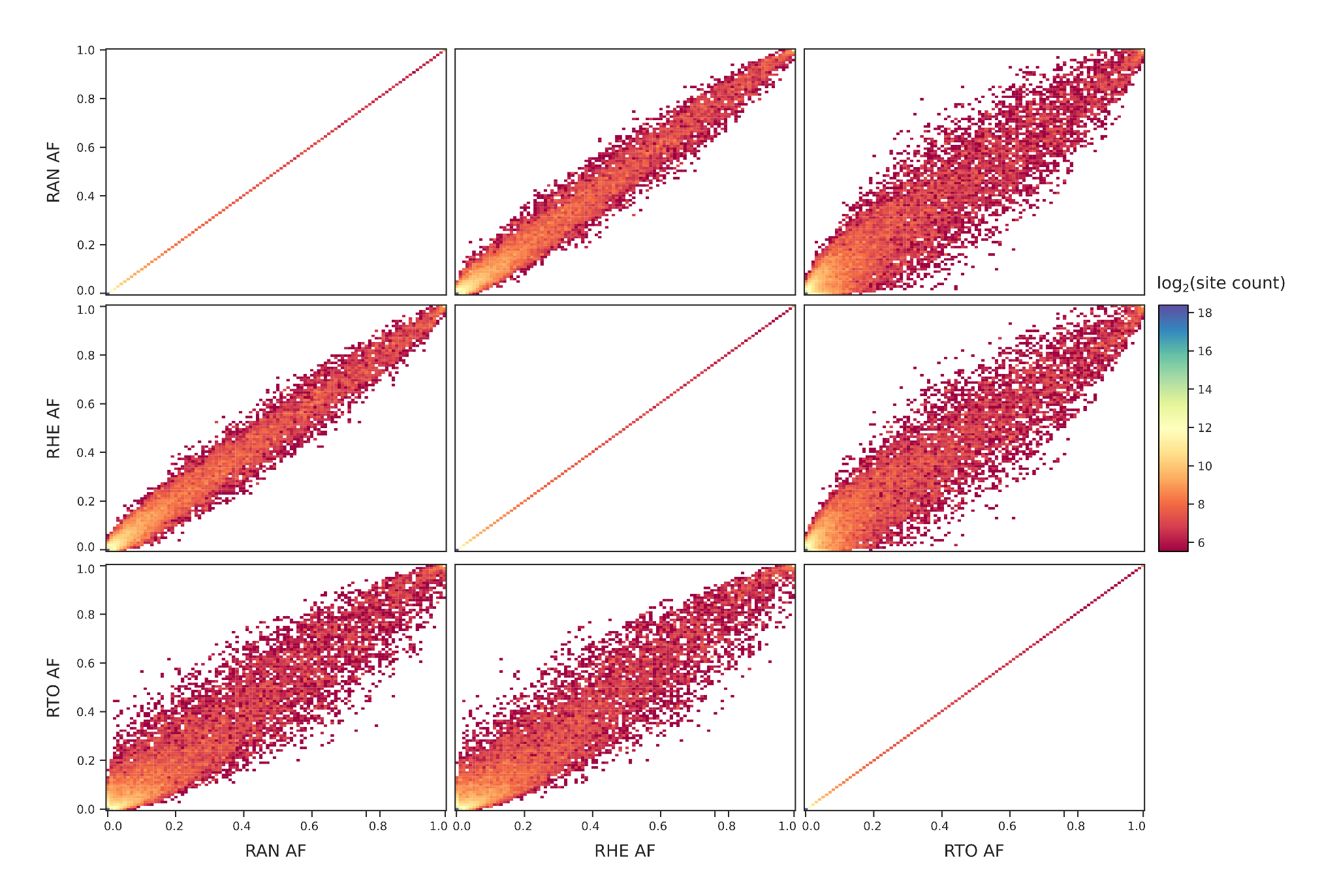
**

**Figure S8.** A scatterplot showing the two-dimensional site frequency spectrum for pairs of Russian population subclusters identified in our analysis. The color of each square corresponds to the log2-transformed number of variant sites with given allele frequency range in the two subgroups.


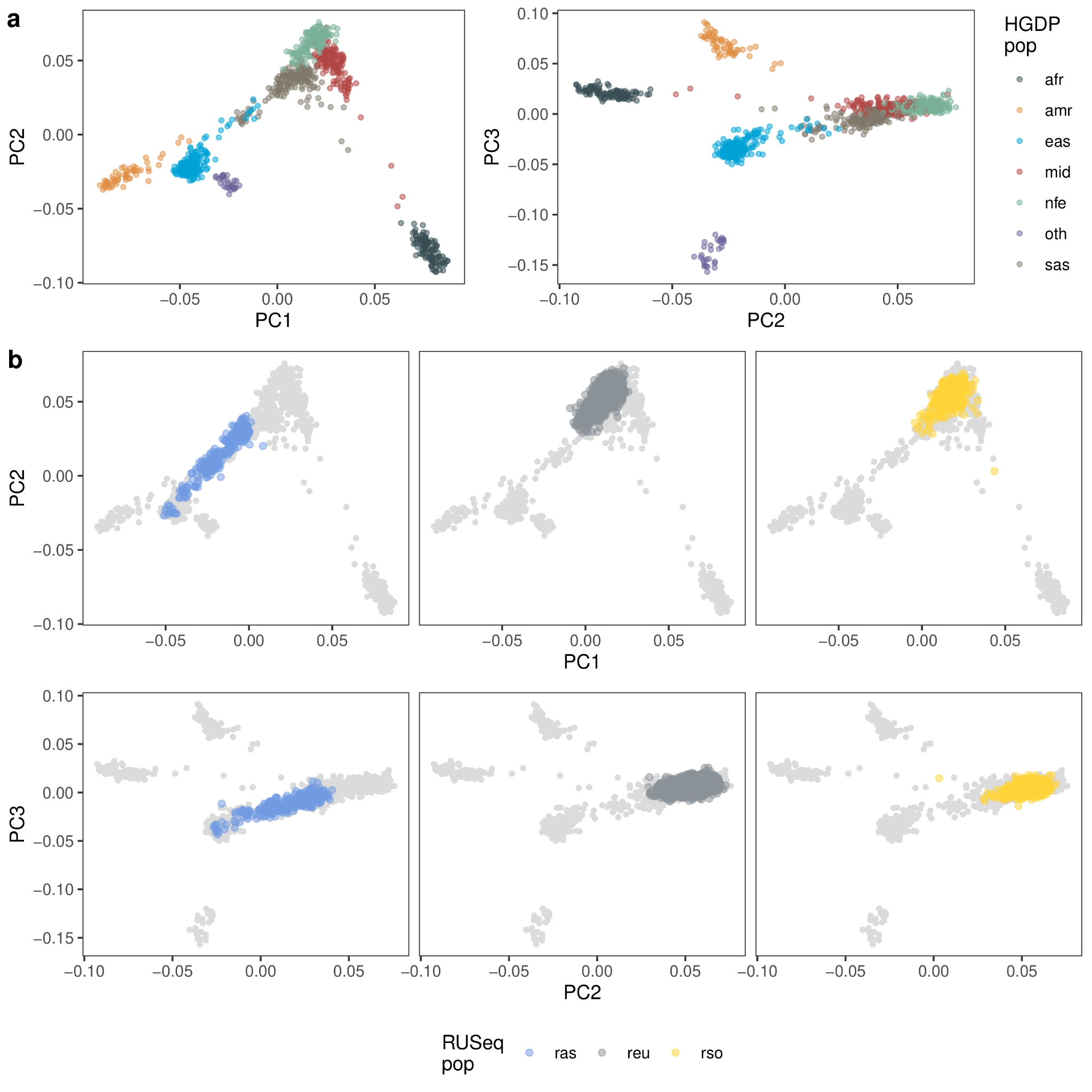


**Figure S9.** A scatterplot of samples from HGDP (a) or RUSeq (b) plotted in the principal component space built using genotypes of the 402 selected HGDP individuals with even geographical representation (see Methods for details). For plots showing RUSeq individuals, positions of HGDP individuals are represented with gray dots. The following abbreviations are used for ancestry groups in 1KGP/HGDP: afr - African, amr - American, fin - Finnish, eas - East Asian, mid - Middle Eastern, nfe - non-Finnish European, sas - South Asian, oth - other. The abbreviations reu, rso, ras stand for the three clusters of individuals in the RUSeq data.


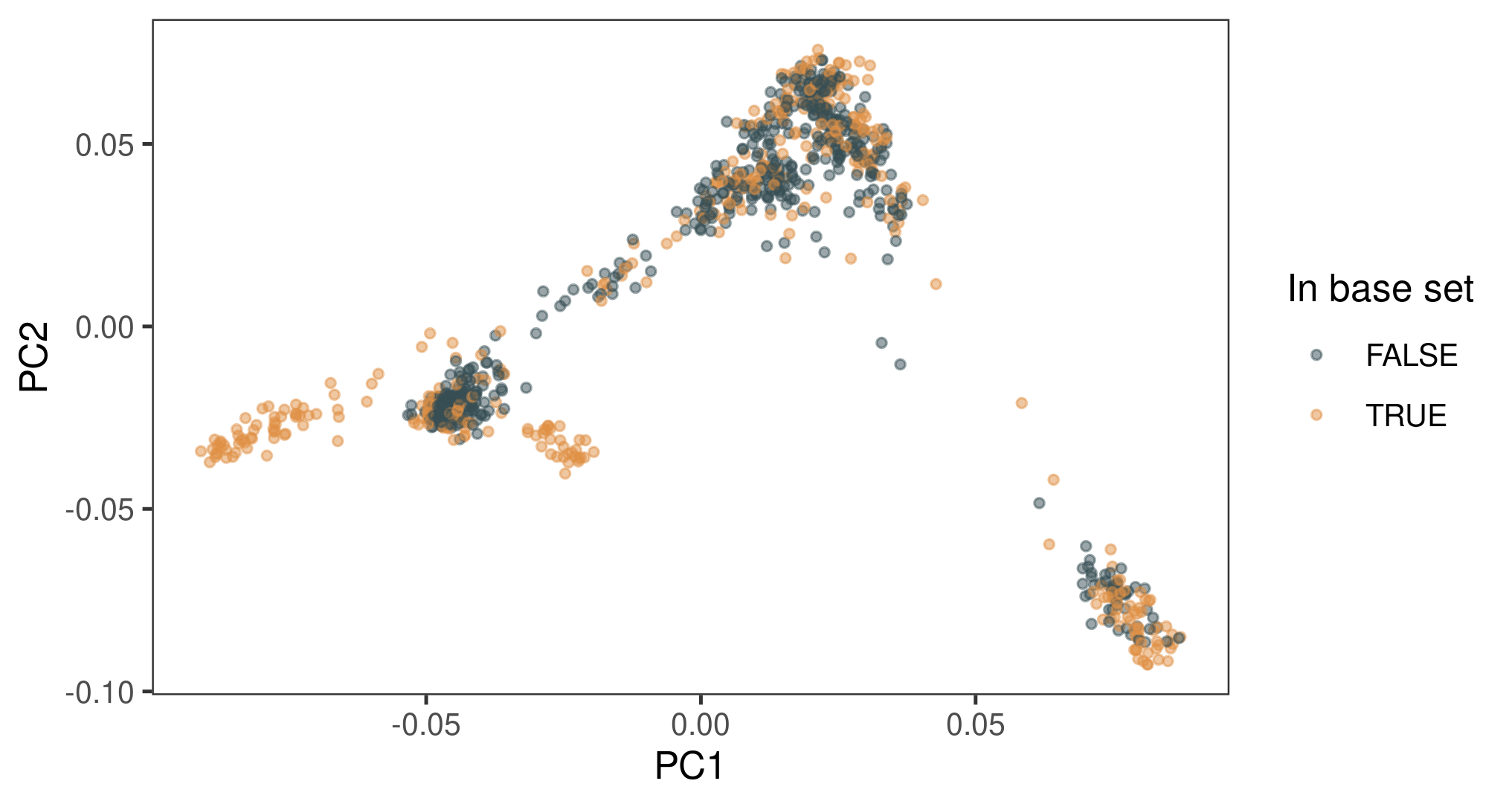


**Figure S10.** A scatterplot showing the individuals from HGDP that were either (i) used to construct PCA or (ii) projected ones in the constructed PC space.

**
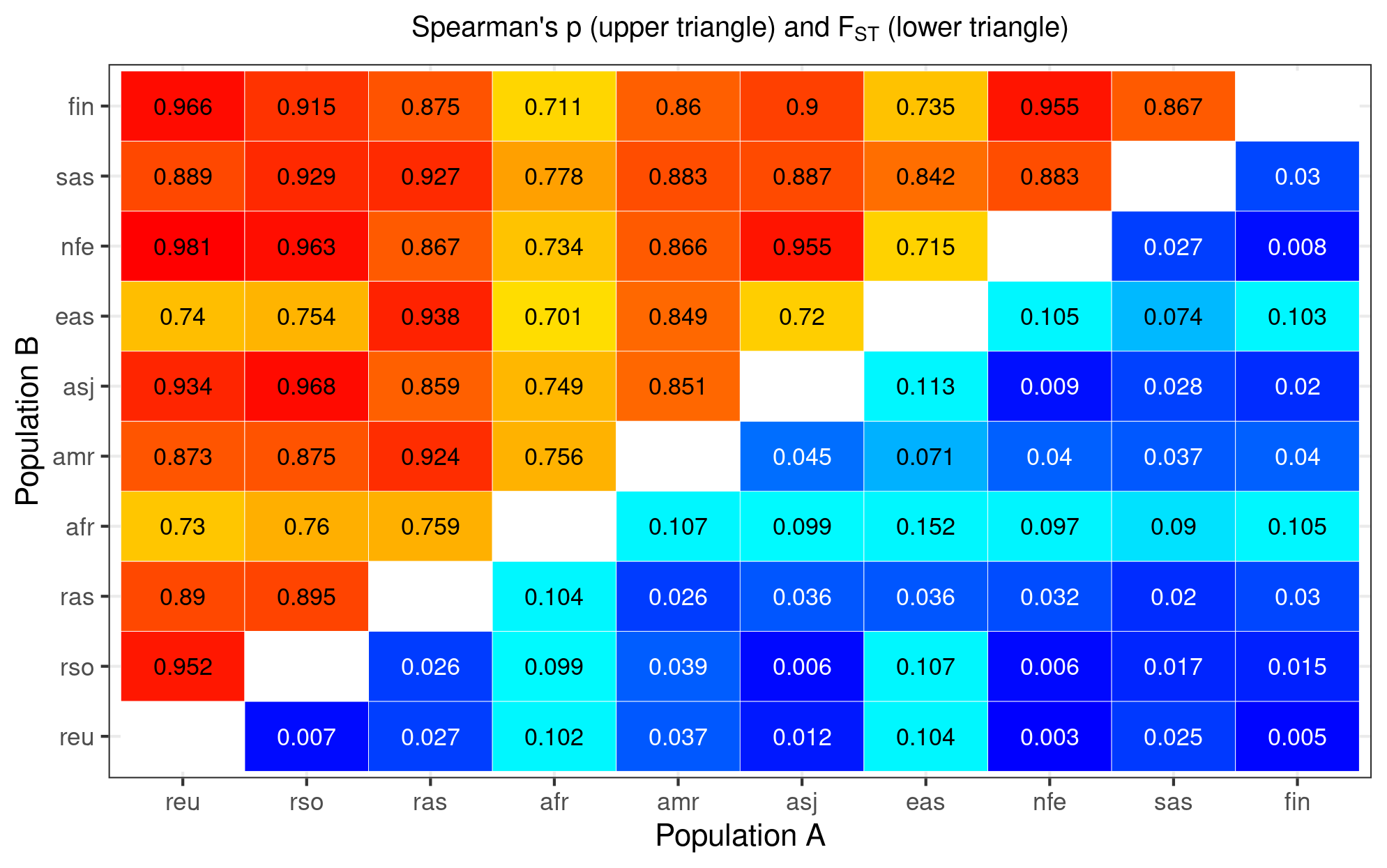
**

**Figure S11.** A heatmap showing Spearman's correlation coefficients between the frequencies of common variants and the *F_ST_* values (estimated using the Weir and Cockerham method) between indicated pairs of populations from gnomAD and RUSeq. AFR - African, AMR - Admixed American, ASJ - Ashkenazi Jewish, EAS - East Asian, FIN - Finnish European, NFE - non-Finnish European, SAS - South Asian. The acronyms reu, rso, and ras stand for the clusters of individuals in the RUSeq dataset.

**
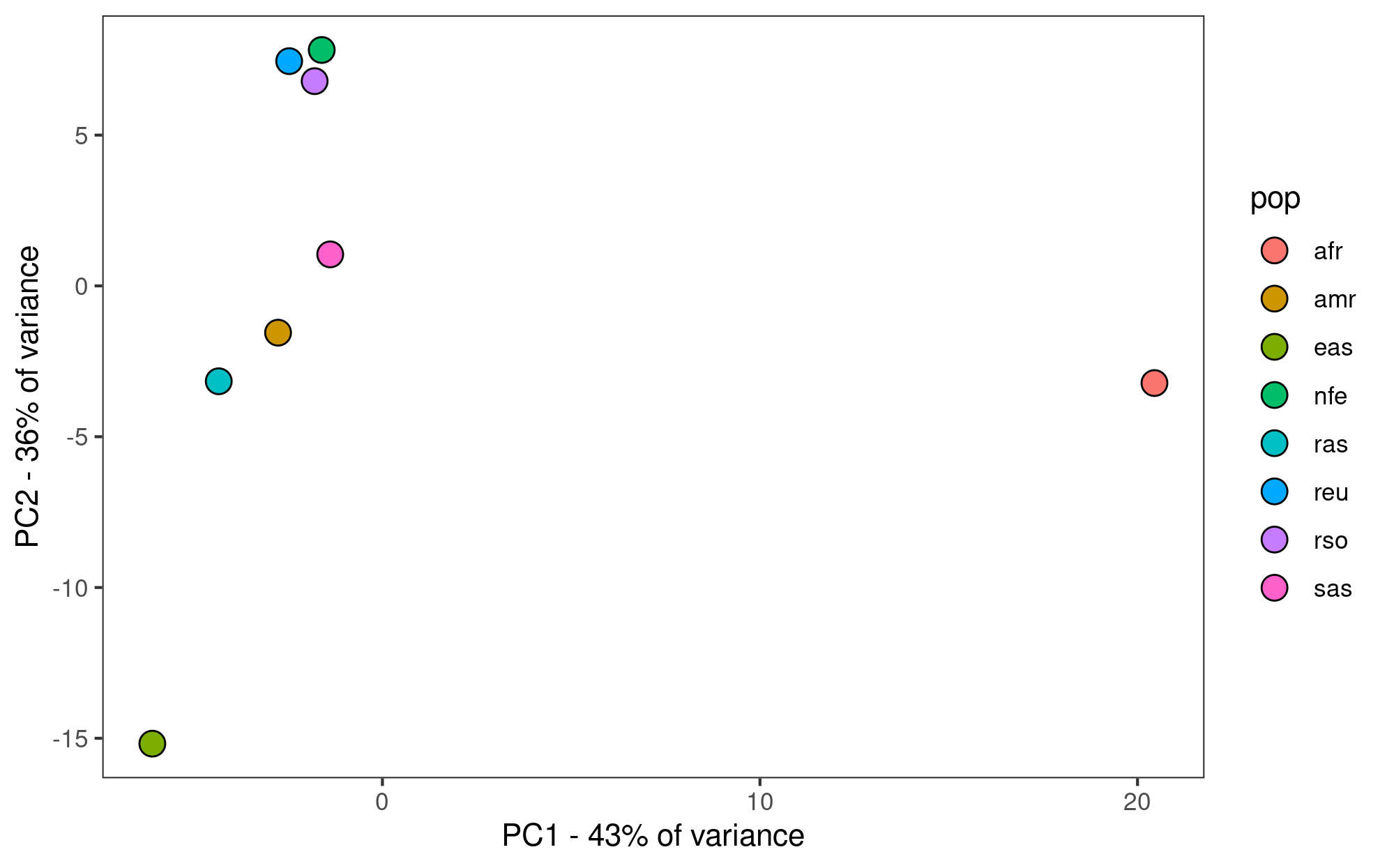
**

**Figure S12.** Principal component analysis of allele frequency data for the set of populations in the Genome Aggregation Database (gnomAD) and RUSeq. AFR - African, AMR - Admixed American, ASJ - Ashkenazi Jewish, EAS - East Asian, FIN - Finnish European, NFE - non-Finnish European, SAS - South Asian. The acronyms reu, rso, and ras stand for the clusters of individuals in the RUSeq dataset.


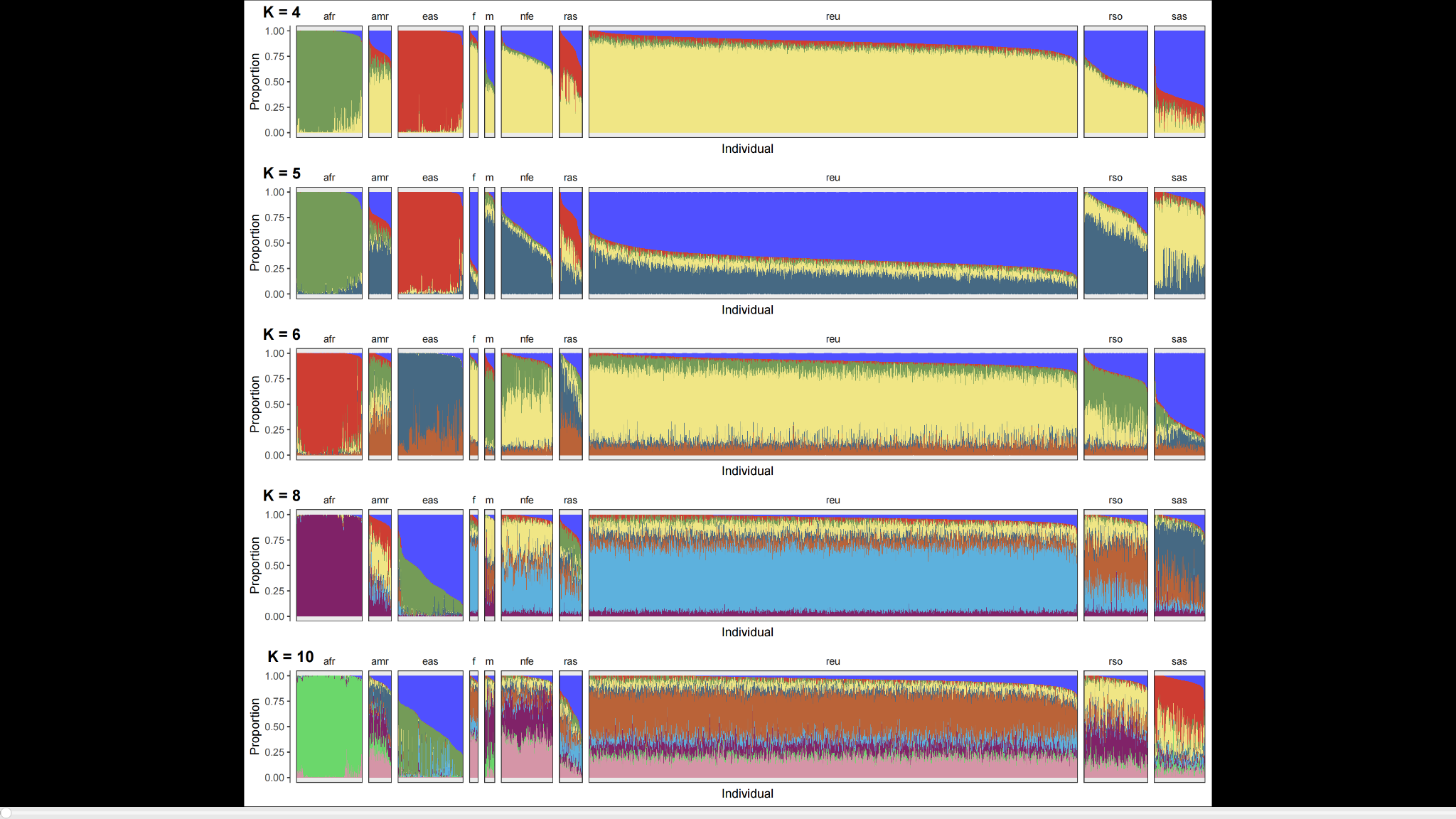


**Figure S13.** Barplots showing the results of ADMIXTURE analysis of the entire set of 10552 individuals from RUSeq, HGDP, and 1KGP with indicated values of *K*. The following abbreviations are used for ancestry groups in 1KGP/HGDP: afr - African, amr - American, f - Finnish, eas - East Asian, m - Middle Eastern, nfe - non-Finnish European, sas - South Asian, oth - other. The acronyms reu, rso, ras correspond to the three clusters of individuals in the RUSeq data.

**
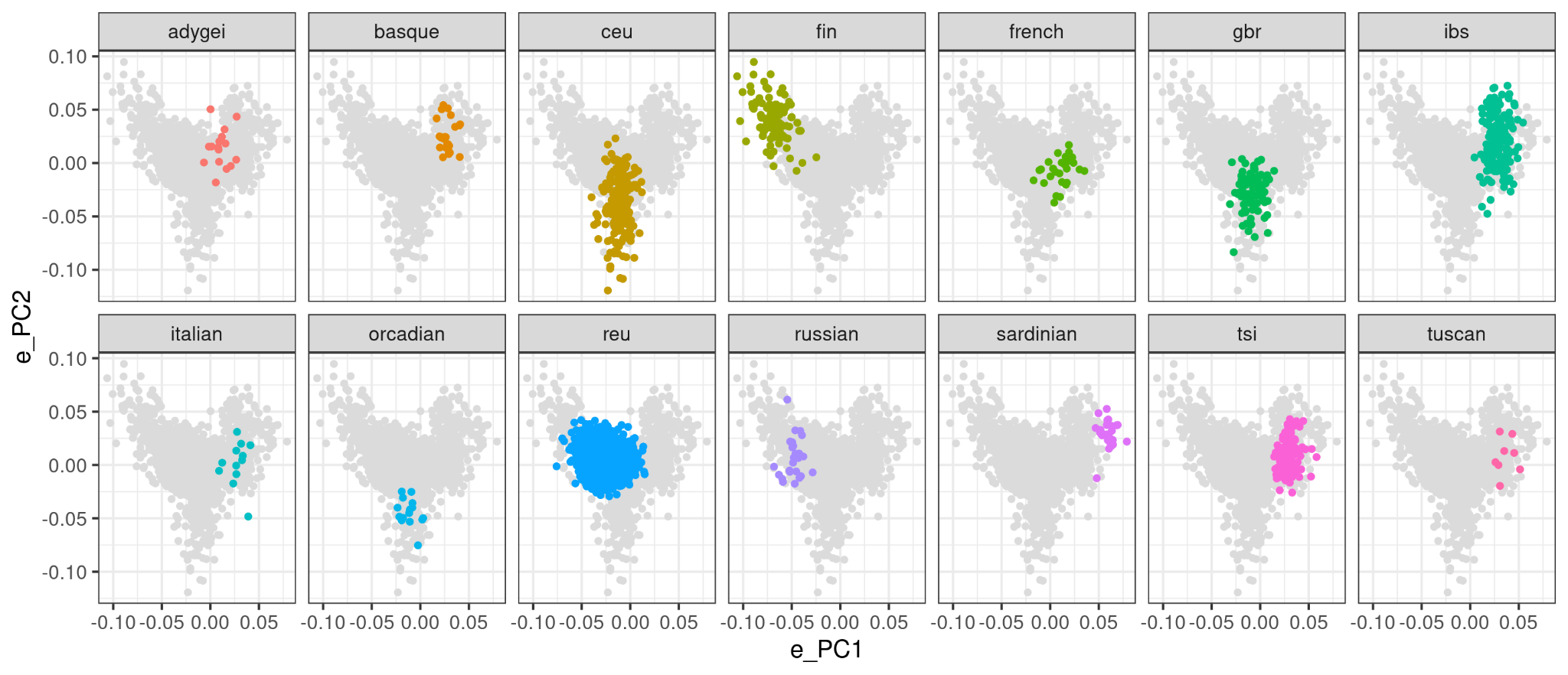
**

**Figure S14.** Principal component analysis of the individuals in the RUSeq European (reu) cluster in the context of HGDP and 1KGP samples of European ancestry. On each subplot, individuals belonging to the indicated subpopulation are highlighted. The following abbreviations are used: ceu - Utah residents (CEPH) with Northern and Western European ancestry; gbr - British in England and Scotland; ibs - Iberian populations in Spain; tsi - Toscanian in Italy.


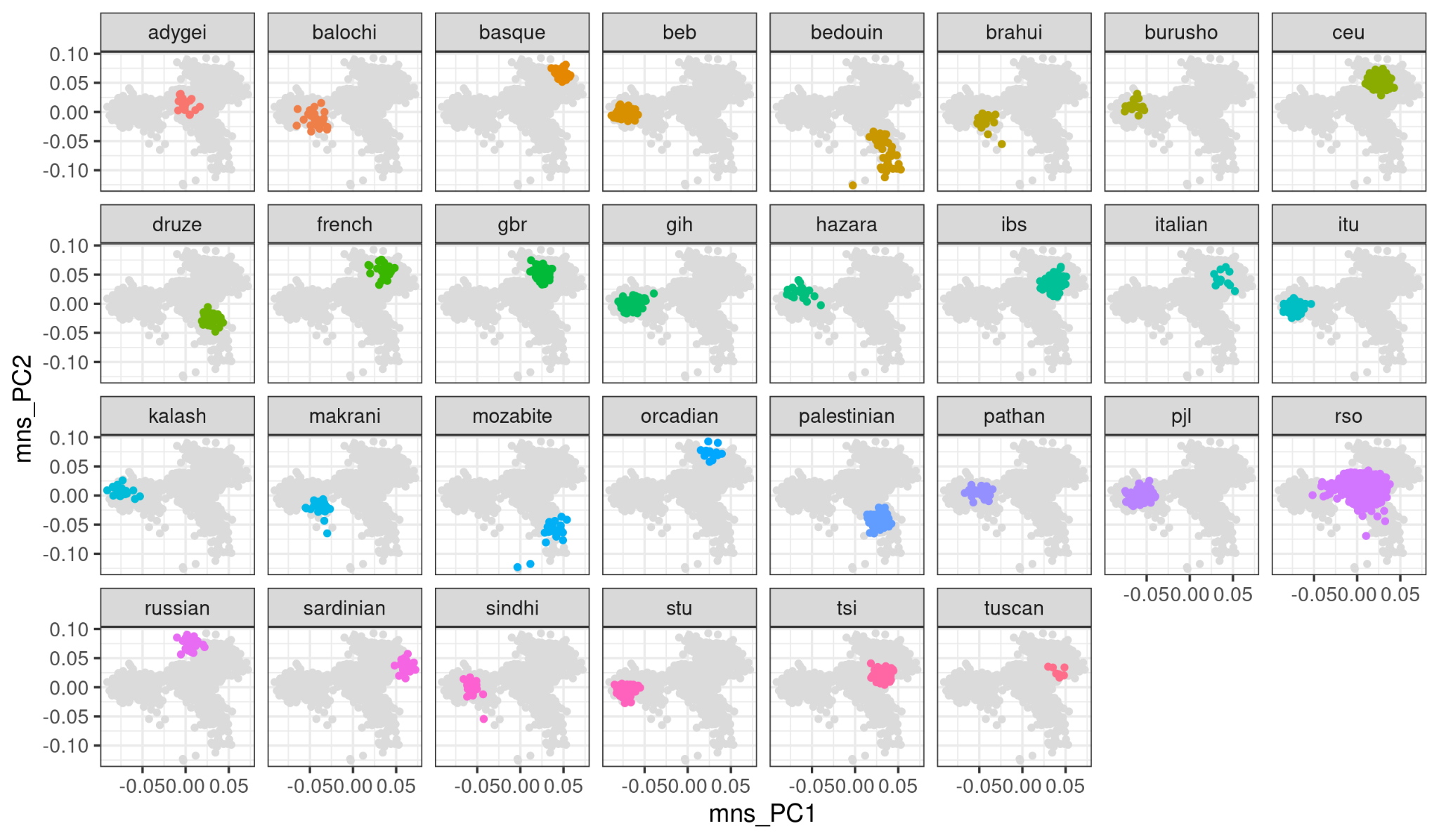


**Figure S15.** Principal component analysis of the individuals in the RUSeq Southern (rso) cluster in the context of HGDP and 1KGP samples of European, Middle Eastern, and South Asian ancestry. On each subplot, individuals belonging to the indicated subpopulation are highlighted. The following abbreviations are used: beb - Bengali in Bangladesh; ceu - Utah residents (CEPH) with Northern and Western European ancestry; gbr - British in England and Scotland; gih - Gujarati Indian in Houston, TX; ibs - Iberian populations in Spain; itu - Indian Telugu in the UK; pjl - Punjabi in Lahore, Pakistan; stu - Sri Lankan Tamil in the UK; tsi - Toscanian in Italy.


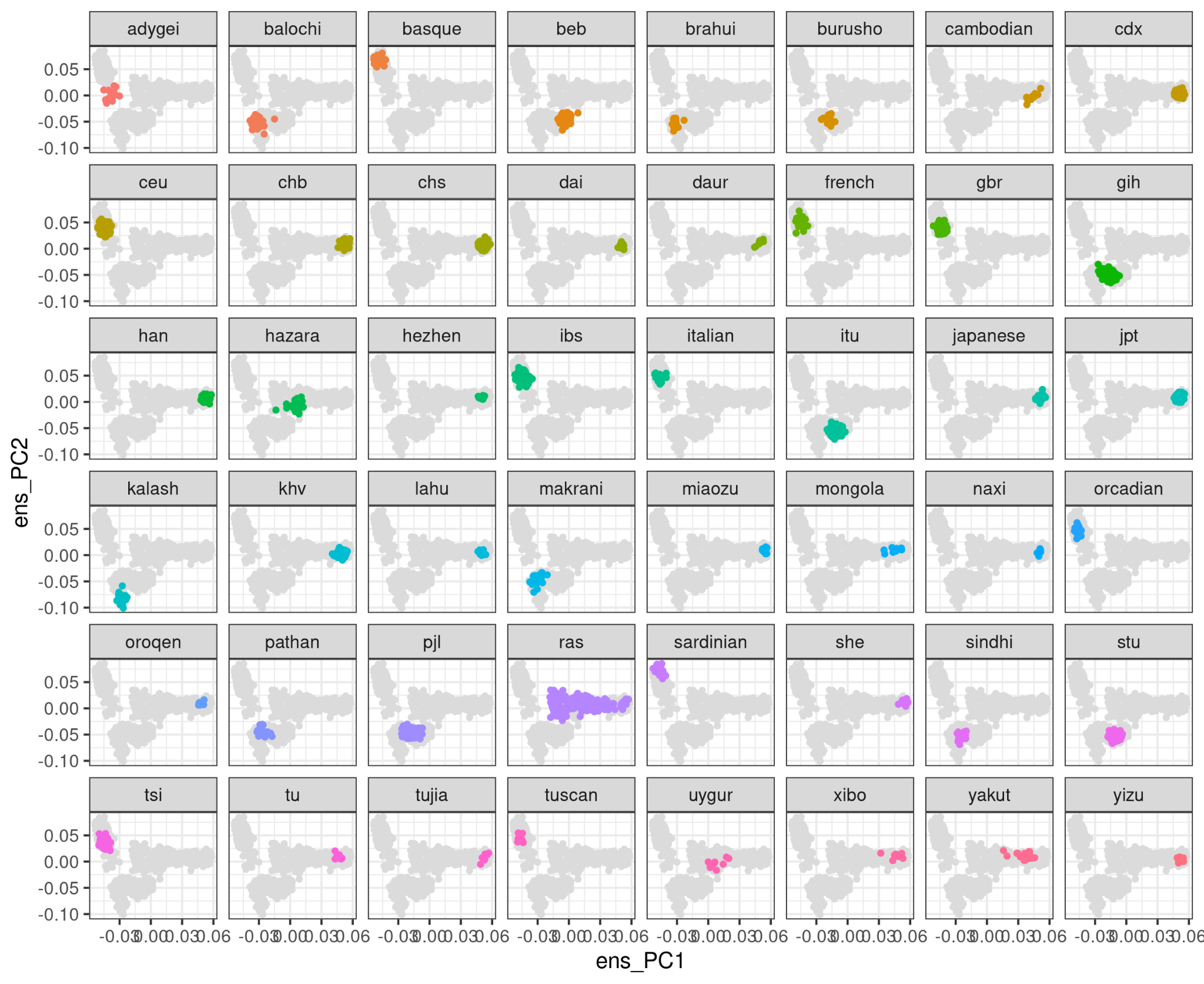


**Figure S16.** Principal component analysis of the individuals in the RUSeq Asian (ras) cluster in the context of HGDP and 1KGP samples of European, East Asian, and South Asian ancestry. On each subplot, individuals belonging to the indicated subpopulation are highlighted. The following abbreviations are used: beb - Bengali in Bangladesh; cdx - Chinese Dai in Xishuangbanna, China; ceu - Utah residents (CEPH) with Northern and Western European ancestry; chb - Han Chinese in Beijing, China; chs - Han Chinese South; gbr - British in England and Scotland; gih - Gujarati Indian in Houston, TX; ibs - Iberian populations in Spain; itu - Indian Telugu in the UK; jpt - Japanese in Tokyo, Japan; khv - Kinh in Ho Chi Minh City, Vietnam; pjl - Punjabi in Lahore, Pakistan; stu - Sri Lankan Tamil in the UK; tsi - Toscanian in Italy.

.

**Supplementary References**

1. Barbitoff YA, Skitchenko RK, Poleshchuk OI *et al.* Whole‐exome sequencing provides insights into monogenic disease prevalence in Northwest Russia. *Mol Genet Genomic Med* 2019;**7**, DOI: 10.1002/mgg3.964.

2. Barbitoff YA, Polev DE, Glotov AS *et al.* Systematic dissection of biases in whole-exome and whole-genome sequencing reveals major determinants of coding sequence coverage. *Sci Rep* 2020;**10**:2057.

3. Li H, Durbin R. Fast and accurate short read alignment with Burrows-Wheeler transform. *Bioinformatics* 2009;**25**:1754–60.

4. Vasimuddin Md, Misra S, Li H *et al.* Efficient Architecture-Aware Acceleration of BWA-MEM for Multicore Systems. *2019 IEEE International Parallel and Distributed Processing Symposium (IPDPS)*. Rio de Janeiro, Brazil: IEEE, 2019, 314–24.

5. Li H, Handsaker B, Wysoker A *et al.* The Sequence Alignment/Map format and SAMtools. *Bioinformatics* 2009;**25**:2078–9.

6. Van der Auwera GA, Carneiro MO, Hartl C *et al.* From FastQ Data to High-Confidence Variant Calls: The Genome Analysis Toolkit Best Practices Pipeline: The Genome Analysis Toolkit Best Practices Pipeline. In: Bateman A, Pearson WR, Stein LD, et al. (eds.). *Current Protocols in Bioinformatics*. Hoboken, NJ, USA: John Wiley & Sons, Inc., 2013, 11.10.1-11.10.33.

7. DePristo MA, Banks E, Poplin R *et al.* A framework for variation discovery and genotyping using next-generation DNA sequencing data. *Nature Genetics* 2011;**43**:491–8.

8. Koenig Z, Yohannes MT, Nkambule LL *et al.* A harmonized public resource of deeply sequenced diverse human genomes. *Genome Res* 2024;**34**:796–809.

9. Price AL, Patterson NJ, Plenge RM *et al.* Principal components analysis corrects for stratification in genome-wide association studies. *Nat Genet* 2006;**38**:904–9.

10. Patterson N, Moorjani P, Luo Y *et al.* Ancient Admixture in Human History. *Genetics* 2012;**192**:1065–93.

11. Alexander DH, Novembre J, Lange K. Fast model-based estimation of ancestry in unrelated individuals. *Genome Res* 2009;**19**:1655–64.

12. Weir BS, Cockerham CC. Estimating F-Statistics for the Analysis of Population Structure. *Evolution* 1984;**38**:1358.

13. Weir BS, Hill WG. Estimating F-Statistics. *Annu Rev Genet* 2002;**36**:721–50.
